# The WGD—A Dataset of Assembly Line Working Gestures for Ergonomic Analysis and Work-Related Injuries Prevention

**DOI:** 10.3390/s21227600

**Published:** 2021-11-16

**Authors:** Christian Tamantini, Francesca Cordella, Clemente Lauretti, Loredana Zollo

**Affiliations:** Research Unit of Advanced Robotics and Human-Centred Technologies, Università Campus Bio-Medico di Roma, 00128 Rome, Italy; f.cordella@unicampus.it (F.C.); c.lauretti@unicampus.it (C.L.); l.zollo@unicampus.it (L.Z.)

**Keywords:** human motion capture, kinematics, working activities

## Abstract

This paper wants to stress the importance of human movement monitoring to prevent musculoskeletal disorders by proposing the WGD—Working Gesture Dataset, a publicly available dataset of assembly line working gestures that aims to be used for worker’s kinematic analysis. It contains kinematic data acquired from healthy subjects performing assembly line working activities using an optoelectronic motion capture system. The acquired data were used to extract quantitative indicators to assess how the working tasks were performed and to detect useful information to estimate the exposure to the factors that may contribute to the onset of musculoskeletal disorders. The obtained results demonstrate that the proposed indicators can be exploited to early detect incorrect gestures and postures and, consequently to prevent work-related disorders. The approach is general and independent on the adopted motion analysis system. It wants to provide indications for safely performing working activities. For example, the proposed WGD can also be used to evaluate the kinematics of workers in real working environments thanks to the adoption of unobtrusive measuring systems, such as wearable sensors through the extracted indicators and thresholds.

## 1. Introduction

The analysis of human motor behaviour is being looked into by the scientific community, as it is transversely in touch with several research fields. Understanding and replicating the human motion behaviour is a common objective among researchers of vary disciplines such as biomechanics, ergonomics, action recognition and computer vision applications up to robotics.

By applying specific protocols, the human body joint trajectories can be reconstructed from raw motion capture data [1]. The obtained information can be used for different purposes, such as performance and motor recovery evaluation, or robot motion control [2,3]. The information gathered from healthy subjects can, for instance, constitute a benchmark for performance evaluations of pathological individuals executing the same tasks [4] or for posture evaluations during working activities. Work-related diseases affecting the musculoskeletal system are very common due to the repetitiveness of certain tasks, to the forces required to complete them and to the posture assumed by the workers [5]. Most of the assembly line working activities carried out in sitting postures are characterized by such negative behaviours. The introduction of a benchmark for the correct postures that people should have during their working activities could help identify kinematics quantities that can be linked to musculoskeletal overloads.

In recent decades, a rich amount of motion capture datasets containing information about human movements have been created and made available to the research community, due to the undisputed utility of human motion data. Some of the most known human motion datasets available in the literature are listed in Table 1. In particular, the table shows the technologies adopted for building the datasets as well as the number of enrolled subjects, performed actions and recorded sequences.

A dataset categorization can be performed on the basis of the technologies adopted for acquiring human movements. Monocular cameras, optoelectronic cameras or multimodal acquisition systems are those mainly used.

Monocular cameras are used to build datasets containing large amounts of videos which are useful for facing the action recognition problem. Some examples are the Human Motion (HMDB51) [6] and the University of Central Florida (UCF101) [7] databases. The Carnegie Mellon University Motion of Body dataset (CMU MoBo) [8] focuses its attention on gait analysis, using data acquired by means of six monocular cameras, whereas the HumanEva dataset [9] addresses articulated human motion and pose estimation.

More accurate human motion kinematic information is retrieved from data acquired by motion capture systems (such as optoelectronic cameras). In the literature, dataset built from this kind of data are proposed. The CMU Graphics Lab Motion Capture dataset [10] is the largest available MoCap dataset and the most used in animation and computer graphics. It contains more than 2000 clips, acquired with a monocular camera and twelve Vicon cameras, about a wide range of actions ranging from locomotion to sports and pantomime. The Hochschule der Medien dataset (HDM05) [11] offers about 70 different motion classes performed by only five actors. Six to twelve cameras of the Vicon optoelectronic motion capture system were used. This dataset was created to facilitate motion generation in data-driven computer animation. The Karlsruhe Institute of Technology (KIT) dataset [12] was introduced to support computer vision and human motion analysis. The Vicon MX motion capture system and a monocular camera collected human motion data. The KIT dataset addressed robot motion generation using imitation learning of their Master Motor Map framework [16,17], which uses a unified reference model of the human body kinematic and dynamic parameters to reproduce motions performed by different subjects.

Due to the different kind of information characterizing the human behaviour, and the increasing availability of different modalities to acquire several types of data, it became more frequent to fuse multimodal data to study different phenomena. Therefore, multimodal datasets have also been developed. The Carnegie Mellon University provides two multimodal datasets, in [13]. The captured actions are related to food preparation. Twelve optoelectronic cameras of the Vicon motion capture system captured kinematic data while other environmental data were acquired by monocular cameras, audio systems, inertial measurement units and wearable devices. The Technische Universität München (TUM) Kitchen dataset [14] collects human motion during the setting of a table. Four static monocular cameras and some RFID tag readings were embedded in the environment. Motion data were extracted from the video using a marker-less motion tracking system proposed by the same authors in [18]. The Berkeley Multimodal Human Action Database (MHAD) [15] dataset contains multimodal information about human motion obtained by the optical motion capture system Impulse, twelve Dragonfly cameras, two depth sensors (i.e., Kinect), microphones and six three-axis accelerometers. Each acquisition device was geometrically calibrated and temporally synchronized with the others. MHAD was specifically built up to test multimodal action recognition algorithms.

From the literature analysis, it appears evident that datasets focused on assembly line working gestures aiming at evaluating and preventing risks of MSDs are missing. With the advent of Industry 4.0 and the increasing incidence of musculoskeletal disorders (MSDs) affecting workers, the availability of information about the characteristic movements of workers could have several advantages. It is worth considering that MSDs represent one of the leading occupational disease worldwide: approximately 31.9% of workers suffer from this pathology [19]. The knowledge of workers’ biomechanics would allow designing better workplaces and, potentially, reduce the occurrence of work-related MSDs due to non-ergonomic postures. A dataset containing the kinematics of assembly line working tasks could represent a useful means for obtaining quantitative indicators to evaluate worker’s postures during their working activities. The detection of incorrect postures could help workers correct their posture and prevent the risk of work-related disorders. Furthermore, in the framework of Industry 4.0, in which humans and robots interact more frequently to fulfill certain tasks, it could be useful to give the robot the knowledge of human motion behaviour as it could allow better safety during the interaction and give them the capacity to move in a human-like fashion [20].

This work introduces the WGD—Working Gesture dataset, a publicly available dataset of assembly line working gestures. A motion capture system, made of optoelectronic cameras, was used to record kinematic data from the upper limbs and trunk of eight healthy subjects performing activities related to assembly line working environments. Handling goods, hammering and screwing activities have been analyzed since they represent the assembly line working tasks with the highest incidence of MSDs, belonging to the manufacturing and agricultural areas [21]. For each repetition, the dataset provides information about the subject’s anthropometry, the 3D coordinates of each marker, joint angles and wrist trajectories with respect to a reference frame positioned on the subject’s shoulder. Moreover, quantitative indicators are also reported in order to provide the means for comparing and evaluating workers’ ergonomic conditions during working activities. These indicators are: the time of execution, the averaged trajectories in both joints and Cartesian spaces with the respective standard deviation, the trunk posture, the inter-subject variability and the dynamic phase plane analysis.

The WGD can be regarded as a tool for researchers that work in the fields of biomechanics, rehabilitation, artificial intelligence and robot control. They can use it for scientific purposes, such as analysing human performance and evaluating ergonomics by extracting new biomechanical and performance indicators, meant to develop and test new machine learning algorithms, or to develop new robot motion control strategies. It aims at providing clinicians/ergonomics evaluators a quantitative tool to support qualitative tests, such as RULA [22], OCRA [5], NIOSH [23], to evaluate motor performance and behaviours. Indeed, the aforementioned qualitative questionnaires, whose definitions are briefly reported in Table 2, are capable of resuming the MSDs risk exposure of a worker but they have to be calculated by an operator observing the working gesture. As a consequence, these methods result in being rater-dependant.

So just as gait analysis, performed with motion capture systems, became a standard procedure for obtaining quantitative information regarding patients as well as healthy people walking, this paper wants to provide normality bands for upper limb motion analysis during their working activities.

The proposed dataset intends to be the first publicly available collection of data about human motion during assembly line working activities. The acquisitions were carried out recruiting eight healthy subjects and asking them to perform working activities related to the assembly line working scenarios where the likelihood of pathologies affecting the muscuoloskeletal system is higher [21]. They are handling goods and assembly line activities (e.g., hammering and screwing). The obtained results can be used as tool for the evaluation of workers’ performance, biomechanics and ergonomics. The averaged trajectories provide information about the reference motion that a worker should follow to correctly execute the activity without overexerting himself/herself. It is obtained since the participants took care about their posture and joint configuration during the recordings, carried out in a highly structured setup. The analysis performed in this paper allows identifying what are the upper limb joints that exhibited wide range of motion for each analyzed working gesture. In addition, the retrieved maximum values assumed by trunk angles can be used to implement monitoring systems capable of delivering alert whenever the user exceeds the computed thresholds. The quantitative assessment of posture and joint angles of healthy participants allows an in-depth understanding of the assembly line working gestures opening the possibility of developing wearable systems that are increasingly aware of the activity performed by the user along with the benchmark to take as reference.

The rest of the paper is organized as follows. Section 2 introduces the WGD: details about the experimental setup, acquisition protocols and dataset organization are provided. Section 3 shows the results of the collected data and Section 4 discusses them. Finally, Section 5 presents conclusions and future work are presented.

## 2. Materials and Methods

### 2.1. Participants

Eight healthy subjects, seven males and one female (mean age 27±4), intact from a neurological and orthopedic point of view, were involved in the study. Both the recruitment of participants and the motion capture acquisitions took place at Università Campus Bio-Medico di Roma, Italy. Motion data were acquired from the subjects performing the identified working tasks. Anthropometric measurements of the right upper limb were collected: 31.25±3.47 cm is the mean length of the right arm while 30.24±1.99 cm is referred to the forearm of the analyzed subjects. All the subjects had a dominant right arm. They also provided written informed consent before participating in this study. The experimental protocol was approved by the local Ethical committee (Comitato Etico Università Campus Bio-Medico di Roma, reference number: 03/19 PAR ComEt CBM) and complied with the Declaration of Helsinki.

### 2.2. Experimental Setup

**Figure 1:**
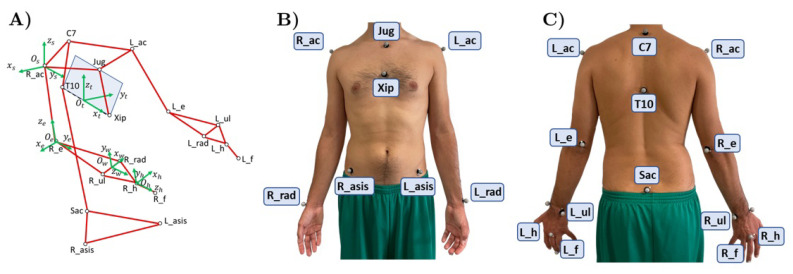
Modified Rab protocol adopted in this study. (**A**) Stick model showing the used protocol for markers positioning. The white dots highlight the marker positioning on the anatomical landmarks used for reconstructing the joint angles while the red lines represent segments connecting the joint centres. The blue plane represents the constructed sagittal plane of the participant. The green frames are used to measure joint rotations. The human model reported in this figure resembles the pose of the subjects at the beginning of each task. A complete list of the abbreviation is provided in Section **Abbreviations**. (**B**,**C**) show a participant equipped with the marker set.

Human motion recordings were performed with an optoelectronic marker-based system, i.e., BTS Smart DX [24]. Eight cameras (resolution 1.4 Mpixel) were geometrically calibrated to capture the motion of reflective elements placed on volunteers’ anatomical landmarks with a frame rate of 60 Hz and an accuracy less than 0.2 mm over a 3 m × 2 m × 2 m area. Nineteen reflective markers were placed on specific anatomical landmarks of the subject’s upper limbs and trunk. The positioning partially followed the methodology adopted by Rab et al. in [25]. The adopted marker set is shown in Figure 1. With respect to [25], the markers placed on the head were removed while three more were added: one marker on the middle finger distal phalanx of both hands, one marker under the xiphoid process (Xip) and one marker at the level of the T10 vertebra. These last two markers were useful to extract trunk posture information, respectively.

### 2.3. Acquisition Protocol

**Figure 2:**
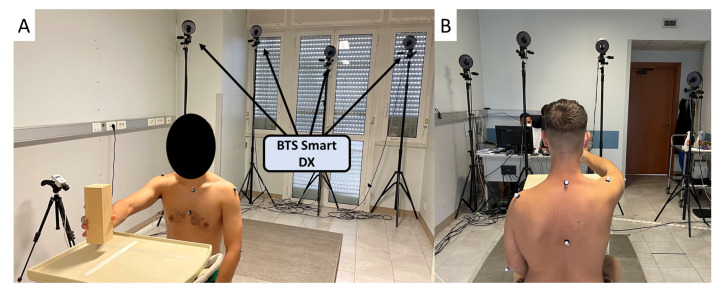
Front and back view of the adopted experimental setup for the acquisition sessions are reported in (**A**,**B**), respectively. The BTS Smart DX optoeletronic system recorded the motion of the subject while performing working gestures.

The subjects were asked to perform gestures related to working activities, i.e., handling goods, hammering, and screwing. Each subject was asked to seat in front of a shelf unit: the table was set at the subject’s shoulder height. The experimental setup used in building up the WGD dataset is shown in Figure 2.

**Figure 3:**
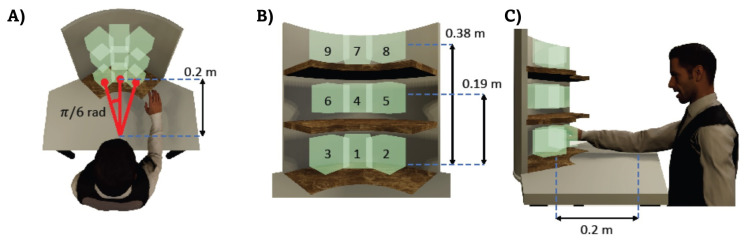
Top, front and lateral view of the shelf unit are shown in (**A**–**C**), respectively. The positions of the targets to be reached are outlined.

The shelf unit used in this experiment was composed of three different levels. The heights of the shelves were 0.19 m and 0.38 m, respectively, from the table. The shelves covered a circular sector of π/3 rad with 0.2 m of radius, to allow lateral load maneuvering and positioning. The target (i.e., the load, the nail or the screw) was initially placed in front of the subject, at 0.20 m distance, while in the subsequent recordings it was moved at π/6 rad and −π/6 rad with respect to its initial position. When subjects were required to perform hammering and screwing tasks, the shelf unit was replaced with wood logs dimensioned in such a way that the nail or the screw were inside the boxes shown in Figure 3. The highlighted positions represent the targets to be reached during the human motion recordings. The working area has been dimensioned by following the ISO 11228-3 directive.

Before the recordings started, the volunteers were instructed on the tasks by an occupational doctor, the number of repetitions to execute and the posture that were meant to be maintained during the recordings to ensure, from an ergonomic point of view, a “safe” task execution. Each task was performed twice, by placing the target at each of the available heights and lateral configurations. Therefore, each of the nine target positions was reached twice.

In the initial position, at the beginning of each recording, subjects were seated in a rest position, with their hands on the table.

### 2.4. Working Task Segmentation

All tasks involve a sequence of consecutive sub-tasks, each with its goals and motion features. The handling goods task was composed of three different sub-tasks in which the volunteers had to reach the box, pick and place it in a specific position and finally return to their rest position (also called homing task). To accomplish the other two working activities, the subject was asked to: (1) reach the working tool (i.e., the hammer or the screwdriver); (2) grasp the object and bring it in contact with the target (i.e., the nail or the screw); (3) perform the working gesture; (4) place the tool on the working table and (5) return to its rest position. Two repetitions of the hammering and screwing sub-tasks were required for each motion recording. The results reported in Section 3 are referred to a single repetition of the hammering and screwing sub-tasks. Several conditions were investigated and recorded for each selected assembly line working gesture. In particular, each subject was asked to perform the three working activities, i.e., handling goods, hammering and screwing, in nine different target positions for two repetitions.

### 2.5. Data Sources and Measurements

Motion Capture data were collected and joint angles were extracted using the software tools provided by BTS Smart DX manufacturer. In particular, the reference frames of the shoulder, elbow, wrist and hand were defined as in Figure 1. Joint angles were extracted from the inverse problem of the XYZ Euler Angles, according to the Rab method [25]. The abduction/adduction (sAA), flexion/extension (sFE) and internal/external rotation (sIE) of the shoulder, elbow flexion/extension (eFE) and pronation/supination (wPS) and the wrist abduction/adduction (wAA) and flexion/extension (wFE) were computed starting from 3D marker coordinates.

In order to compare all the trajectories performed by the subjects, the trajectory dependency from the starting position was removed. Hence, the Cartesian trajectories, i.e., the wrist displacements, performed by the subjects were computed as

(1) $$p\left(t\right)=p_{w}^{s}\left(t\right)-p_{w}^{s}\left(t_{0}\right)$$

where $p_{w}^{s}$ is the position of the wrist with respect to the shoulder frame and *t* and $t_{0}$ are the current and sub-task initial time, respectively.

The posture of the participants was also assessed. In order to compute the trunk reference frame, the sagittal plane was defined as the plane passing through the three markers Xip, T10 and Jug. The trunk reference frame center $O_{t}$ was located in the middle point between the Xip and the T10 markers. The $x_{t}$ was modeled as the unit vector connecting the T10 and the marker on the xiphoid process. The $y_{t}$ axis was computed as the unit vector outgoing the sagittal plane, pointing toward the left side of the body. The $z_{t}$ axis, included in the sagittal plane, was computed as the cross vector between $x_{t}$ and $y_{t}$ axes.

The relative rotations of the trunk frame in a certain time stamp with respect to the starting one (recorded in t=0), was computed to monitor the trunk rotations. Given the rotation matrix $R_{T}^{GRF}\left(t\right)$, indicating the rotation of the trunk reference frame with respect to the global reference frame (GRF) at time *t*, the rotation of the trunk $R_{T}\left(t\right)$ during time is expressed with respect to the $R_{T}^{GRF}\left(t\right)$ computed at the time $t_{0}$, i.e., the time in which the recording began, by the following equation

(2) $$R_{T}\left(t\right)={\left(R_{T}^{GRF}\left(t_{0}\right)\right)}^{-1}\cdot R_{T}^{GRF}\left(t\right).$$

The XYZ Euler Angles extracted from $R_{T}$ provide the lateral bending (tLB), flexion/extension (tFE) and torsion (tT) of the trunk, respectively.

Tracked raw data were filtered with a 4th order low-pass Butterworth filter, with a cutoff frequency $f_{cut}$ of 3 Hz. Such value was chosen because noise and motion artifacts can be found at higher frequencies. It was confirmed by the results of a residual analysis performed on the collected data [26]. The $f_{cut}$ of 3 Hz led to a residual RMS of $\left(5.9\pm 1.6\right)\times 10^{-4}$ m for the hammering, i.e., the most impulsive task. Such a small residual confirmed that the chosen value of $f_{cut}$ is suitable for all the tasks, being the handling goods and the screwing tasks slower than the hammering. Furthermore, anthropometric measurements were collected for each subject. These properties were obtained manually by measuring arm and forearm lengths with a tape.

In order to test the inter-subject variability, the deviation between the wrist trajectories was also assessed. The following equation was implemented to quantify such metrics:

(3) $$V=\sqrt{SD_{x}^{2}+SD_{y}^{2}+SD_{z}^{2}}$$

where SD stands for the standard deviation along one Cartesian axis. The same metrics was also used in [27]. The inter-subject variability of other angular quantity, i.e., upper limb joints and trunk compensatory movements, were assessed by standard deviations.

The selected assembly line working gestures were also analyzed in the phase plane. Phase plane analysis has the potential to be a powerful tool to diagnose motor disorders: graphically representing the velocity $\dot{p}\left(t\right)$ against the displacement p(t) allows the appreciation of the movement periodicity [28]. In fact, rhythmic tasks are represented by closed cycles in the phase plane: they follow a certain temporal evolution before returning in the starting point (i.e., starting position and zero velocity).

Finally, an additional recording is collected from a male volunteer (age 25) in order to evaluate the capability of the data stored inside the WGD to serve as a tool for posture evaluation. The participant performs a handling goods task in which he moves moving the box from the starting position toward the position number 9 (Figure 3), without any external advice about how to execute the task in an ergonomic manner. This task has been chosen because it is the most critical one: it requires both cross-body motion and high shoulder elevation, representing risky postures according to the RULA checklist. The subject posture is monitored, in terms of trunk rotations, and the resulting angles are compared with those reported as maximum values stored inside the WGD.

### 2.6. Dataset Organization

The dataset is a folder structure whose contents are organized as follows. The *Kinematic_data* folder includes a file named *Anthropometric_measurement.txt* containing a 8×2 matrix where the rows represent the subjects while the columns contain the length of the measured arm and forearm, respectively. A sub-folder for each working action, i.e., handling goods, hammering and screwing, is also supplied. Several files are provided for each motion capture recording of each subject.

- Raw data: the raw data collected from the motion capture system is reported in the “*.../raw_MoCap.txt*” file.
- Marker positions: the three-dimensional position of each marker placed on the body of the subject are stored inside the “*.../points.txt*” file.
- Joints Trajectories: “*.../joints.txt*” file contains the trajectories executed by the joints of the right upper limb. In particular, the following degrees of freedom (DoFs) are listed: shoulder internal/external rotation, adduction/abduction and flexion/extension, elbow flexion/extension and prono/supination, wrist adduction/abduction and flexion/extension.
- Trunk Rotations: “*.../trunk_rotations.txt*” includes the Euler angles of the trunk with respect to the starting position acquired at t=0 s. The reported information is about the trunk flexion/extention, lateral bending and rotation.
- Cartesian Trajectory: the wrist displacements p(t), computed as reported in Equation (1), are stored in the “*.../Cartesian.txt*” file.
- Task Segmentation: “*.../task_segmentation.txt*”. An analog trigger signal was synchronized and recorded with the motion capture system to identify the temporal transition from one sub-task to the subsequent one. This file contains an integer variable that increases once the subsequent sub-task begins.

The WGD is a continuously growing structure that will be gradually enriched with new kinematic data as soon as they will be acquired.

## 3. Results

The eight enrolled subjects performed the assembly line working gestures with their personal motion style, while still paying attention to maintain the most ergonomic posture as possible. Consequently, they accomplished the required tasks in different times. The time they spent to perform the working action is reported in Table 3.

**Figure 4:**
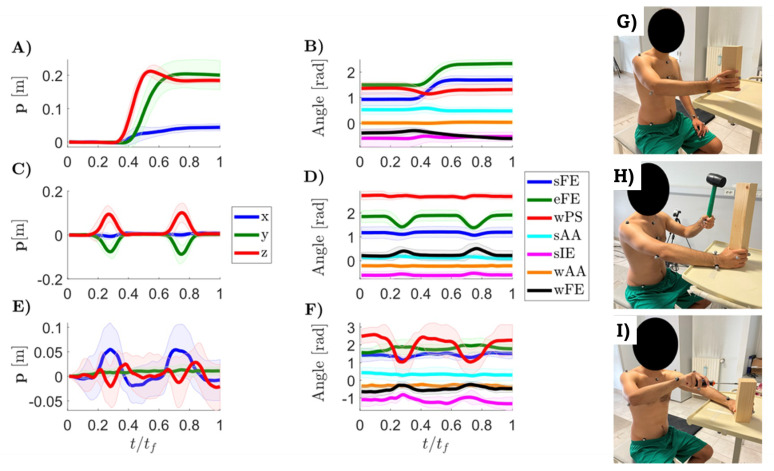
Mean (solid lines) and standard deviation (shaded area) of both Cartesian trajectories (**A**,**C**,**E**) and joints (**B**,**D**,**F**) of the WGD. The time (*t*) is normalized with respect to the time needed to complete the movement ($t_{f}$). These plots were obtained by averaging all the collected trajectories of all the enrolled subjects for all the executed repetitions, retrieved at one specific target position. In particular, the (**A**,**B**) is referred to handling goods task, (**C**,**D**) to hammering and (**E**,**F**) to screwing. For both the hammering and screwing are reported two repetitions. Handling goods, hammering and screwing real working activity executions are reported in (**G**–**I**), respectively.

In Figure 4, the temporal evolution of both the monitored Cartesian displacements and joint angles of the right upper limb during the execution of the working gestures are shown. On the x-axis, the time (*t*) normalized with respect to the time needed to complete the movement ($t_{f}$) is shown. Figure 4A,B are referred to the handling goods task, Figure 4C,D are for the hammering while Figure 4E,F represent a screwing task. For the last two working tasks two repetitions are reported. In particular, mean and standard deviations, calculated using the two repetitions of each subject for each target position, are evaluated for each measurement.

The reported trajectories are referred to the target position number 4, 8 and 4 (see Figure 3) for the handling goods (Figure 4A,B), the hammering (Figure 4C,D) and the screwing (Figure 4E,F) tasks, respectively.

The trunk posture of the subjects was assessed by measuring its three angles related to antero-posterior and medio-lateral bending and torsion. Table 4, reports the interquartile ranges of the maximum/mean value and standard deviation of the trunk angles collected in the recordings.

Inter-subject variability was also assessed. Table 5 shows the maximum values returned by Equation (3), for each task and target position. The inter-subject variability of the recorded movements at the upper right limb joints is highlighted by the standard deviation shown in the right column of Figure 4. Postural compensatory movements reported in Table 4 include also the variability of the maximum and mean angles for each trunk possible rotation.

**Figure 5:**
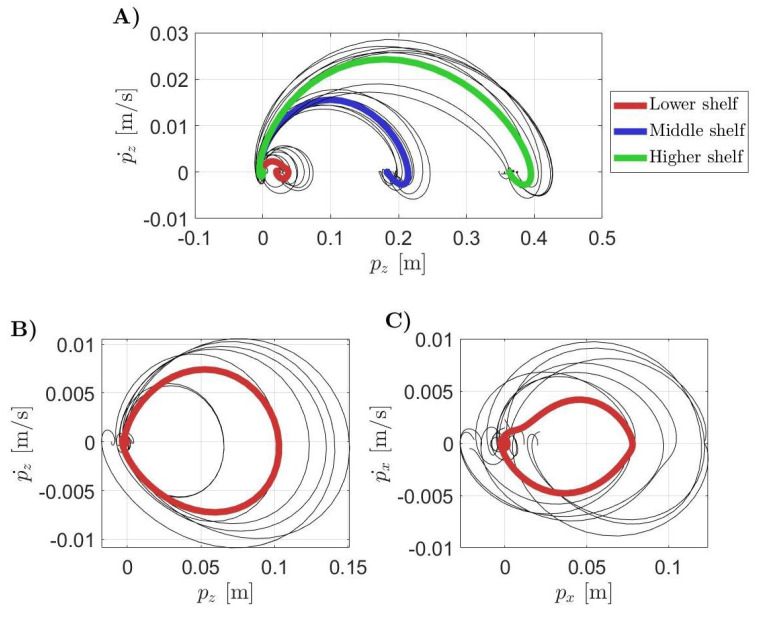
Phase plane plots for the working gestures of the WGD. The plot (**A**) is referred at the handling goods task: the higher the shelf, the higher the displacement from the origin. Plots (**B**,**C**) are about the rhythmic tasks, i.e., hammering and screwing. In particular, the dynamic evolution of the hammering sub-task is reported on the left while there is the screwing on the right. The coloured and gray lines represent the mean phase plane trajectory and the single recorded trials, respectively.

Phase plane plots are shown in Figure 5. The thin grey lines are all the collected data whereas the thick lines are the averaged trajectories. In Figure 5A, the pick and place behaviour of the handling goods task in the phase plane of the motion recorded toward the targets number 1, 4, and 7 of Figure 3, is highlighted. In this plot, the dynamic evolutions of the displacement p(t) along the Z axis according to the different target positions are distinguished. The red line stands for the lower shelf, blue for the middle one and green is for the highest one. Figure 5B,C show the phase planes for the hammering and screwing working gestures executed with the nail and the screw, respectively, located in the target position number 4 of Figure 3. The displacements p(t) along the Z and the X axes were studied, respectively, for the hammering and the screwing sub-tasks since they are the most representative ones.

**Figure 6:**
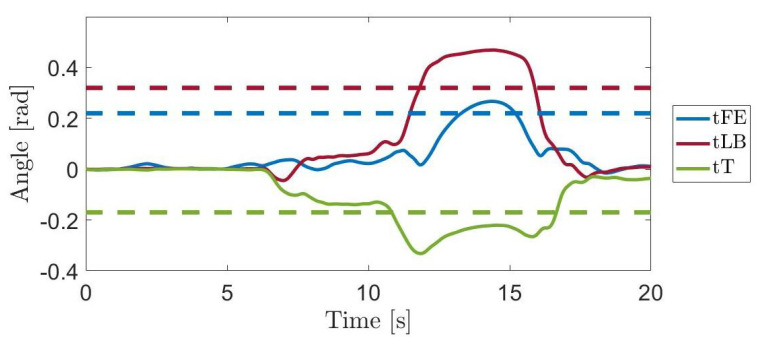
Rotations of the trunk recorded during the test trial. The dashed horizontal lines stand for the maximum threshold values suggested by the WGD.

The trunk rotations collected during the additional recording to assess the applicability of the WGD as a tool for posture evaluation are shown in Figure 6. In particular, trunk angles exhibited by the volunteer during the execution of a handling goods task are reported. In the figure, the dashed lines are the maximum values of the trunk rotations reported in Table 4. Such threshold values are 0.22 rad for the tFE, 0.32 rad for the tLB and 0.17 rad for the tT degrees of freedom.

## 4. Discussion

In this section, the quantitative indicators introduced in Section 2, are used to carry out a kinematic evaluation of the analyzed assembly line working gestures and to demonstrate the capability of the WGD to be used as a quantitative tool to support qualitative checklists. In view of the results presented in Section 3, some consideration can be made to let WGD users able to draw human ergonomics evaluations.

From Table 3 it can be understood that the activity requiring the shortest execution time was the handling goods: it took 3.17±0.98 s. On the other hand, the single repetition of the hammering and the screwing tasks were accomplished in 3.62±0.83 s and 3.68±0.82 s, respectively. This because these gestures needed more accuracy than the other.

The motion parameters presented in Section 3, and available in the WGD, can be used as normality bands for analyzing the data acquired from workers to assess in real time their motor behaviours. The Cartesian trajectories and joint angles reported in Section 3 are representative of all the positions investigated during the recording sessions. From the visual inspection of the averaged trajectories, the following aspects are worth to be highlighted.

The handling goods task is shown in Figure 4A,B. In the Cartesian space, the subject wrist performed a minimum-jerk-like trajectory in XY plane while the Z coordinate exhibited a characteristic sopra-elevation before the goal was reached. This sub-task allows appreciating the excursions of the joints of the upper arm according to the target position: both the shoulder and the elbow exhibited different levels of extensions. When the target position was in higher shelves, the joint extension angles were bigger. At the same time, moving the target along the lateral direction, the subject’s shoulder needed different level of abductions.

The wrist displacement during the hammering task, showed in Figure 4C, was a bell-shaped trajectory in both Y and Z directions. In this particular example, the X component was still when compared to the others, meaning that the participants loaded and hit in their sagittal plane. Whenever the target position crossed the other side of the body, the X component also assumed the same bell-shaped aspect because the overall motion was performed inside a rotated plane. The hammering of the nail showed different motion patterns according to the target position also in the joint space (Figure 4D). Peculiar behaviours could be distinguished between the trials. The sAA was fixed by the subjects during task execution, according to the nail position. The higher the shelf, the lower the abduction angle was required. The most involved degrees of freedom in this task were eFE and wFE, as they were solicited in all the trials. In particular, high wFE range of motion (0.49±0.35 rad) was required for all the trials performed at the lower and middle levels. When the nail was put frontally to the subject, the observed wrist rotations were smaller than the eFE RoM (0.38±0.24 rad).

The screwing Cartesian displacements and joints angles are reported in in Figure 4E,F. In Cartesian space, it was evident that the wrist performed a rotation in the XZ plane, around the Y axis. Regarding the joints angles, it can be noted that the wrist angles, in particular the wPS and wFE, are the one exhibiting the highest range of motion.

The performed analysis allowed identifying the joints working far from the neutral position for each task. In particular, the angles of the shoulder articulation reached high values during handling goods. The elbow and the wrist were mainly involved in the hammering task, whereas the screwing required high wrist angular displacements. These considerations are useful to adopt more focused monitoring actions during the working activities to detect beforehand the incorrect gestures and postures and, consequently prevent MSDs. Wearable monitoring devices could in fact be reduced in number and located in specific anatomical locations.

The postural compensations of the subjects were assessed during the execution of each working activity. Table 4 presents the interquartile ranges of the maximum trunk rotation for each monitored degree of freedom. The tFE in the handling goods task showed very little rotation. Otherwise, when the target position required cross-body motions, the trunk distinctly bended and rotated. These results are in agreement with the findings in [29] in which upper limb kinematics and trunk compensatory motions were characterized during functional tasks.

On the other hand, the subjects exhibited higher antero-posterior bending during hammering and screwing tasks. tLB and tT were less prominent than the ones observed in the handling goods task.

This points out that the trunk motion is particularly wide during most of the working activities. Cross-body motions as well as other functional activities can excessively load workers’ spine. A specific program addressing this issue is therefore necessary.

Results about inter-subject variability, measured with Equation (3), are shown in Table 5. In particular, for each target position, the maximum value of the computed variability is reported. The highest variability obtained for the handling goods task was 0.13 m. The target positions which amplified such variability were the ones involving the higher shelf. In the calculation of the total variability, the highest component was always the Y (as shown in Figure 4A), that represents the distance in depth of the target with respect to the subject. It is due to the fact that the volunteers were asked to place the load at the target position on the shelf but the distance of the shelf with respect to the subjects was not a priori fixed. Hammering was the working activity with the highest variability. The amplitude of the hammering was very different from one subject to another, according to their personal motion style. On the other hand, the screwing task reported lower variability due to the limited movement executed in the Cartesian space.

To appreciate movement periodicity, a phase plane analysis was carried out. This graphical representation allows identifying rhythmic patterns from an executed motion. The pick and place sub-task of the handling goods, shown in Figure 5A, was discrete. The gesture ended when the target position was reached. The higher the shelf, the higher the required displacement was from the starting position. On the other hand, hammering and screwing activities showed a degree of cyclicity in Figure 5B,C. In fact, in the phase plane the trajectories started from the point of initial position and zero velocity to return to the same condition. The hammering gesture presented a repeatable shape in the phase plane with the only variability in the amplitude of the movements. For the screwing sub-task, on the other hand, the shape was not so well defined even if the cyclical behaviour of the movement was still evident. A residual displacement of about $3.1\times 10^{-4}$ m could be seen in the averaged trajectory of the y coordinate in the phase plane. A possible explanation of this residual is that at the end of each manual screwing procedure it was not possible to guarantee that the volunteers returned perfectly to their initial positions. This did not happen for the hammering task as this gesture began and ended with the hammer positioned on the head of the nail.

This analysis was important to find out the most repetitive tasks since they are the most dangerous ones. Hammering and screwing were the identified ones, therefore specific interventions, such as more frequent scheduled pauses and stretching exercises are recommended.

Finally, the test trial points out the paramount role of postural training to obtain an ergonomic posture during working activities. Workers can benefit from monitoring systems returning alerts whenever they exceed the reference values introduced in this work. The collected data can serve as a quantitative tool to define normality bands for upper limb motion evaluations since the recordings are carried out in structured and controlled conditions.

## 5. Conclusions

In this paper, a dataset of human assembly line working gestures was presented and made available to the research community.

In order to build the proposed dataset, eight healthy participants were recruited. They were asked to perform the working activities with the highest incidence of MSDs, i.e., handling goods, hammering, and screwing. Joint angles of the upper limbs in addition to the wrist Cartesian position with respect to the shoulder frame were obtained during the execution of each task by means of a motion capture system. A folder tree structure has been chosen for the dataset in order to better access each dataset element.

Due to the wide range of provided data, the dataset presented in this paper can be easily exploited in several research fields. Several analyses were conducted on the collected data to prove the WGD’s potentialities as a tool for kinematics evaluations.

In this paper, averaged trajectories were presented and distinctive motion features were computed. These measurements, i.e., the mean trajectories, standard deviations, trunk rotations, inter-subject variability and phase planes, were useful to determine and understand the human motion patterns used in performing assembly line working gestures. The variability observed between subjects was very small considering the lack of strict constraints during the recordings of the working tasks. This allowed the definition of reference trajectories of the analyzed gestures and highlighted how the WGD can serve as a benchmark for kinematics evaluation of workers.

The performed analysis introduced useful indices and thresholds for monitoring systems in the working environments to detect incorrect task executions and improve work-related injury prevention. Joints exhibiting the highest range of motion during working activities were identified to provide guidelines for addressing workers’ safety. The information contained in the WGD were collected from healthy participants with a highly structured setup and with gold standard motion capture devices since we wanted to retrieve data with low measurement errors in order to build up the WGD. When monitoring real workers in their working environment, also other motion analysis systems (such as wearable [30] or visual [31] sensors) can be adopted. Since the database is made of kinematic information, comparisons can be easily made among motion captured data with the reference values proposed in the dataset. The WGD can therefore represent a quantitative tool to support clinicians and ergonomics evaluators.

Future work will be devoted to increasing the number of enrolled subjects and the number of the analyzed working gestures. In addition, the participants kinematics will be recorded while performing the same assembly line working gestures in an erect posture. This way, other important measurements such as the centre of pressure could be taken into account. Moreover, participants affected by MSDs could be enrolled in order to study how the kinematics is altered according to the pathological condition.

## Figures and Tables

**Table 1:** Overview of human motion datasets available in the literature. The table lists the technologies used in the dataset building. In the “**Monitored Body Part**” column, NA stands for “Not Applicable” since those datasets do not provide Motion Capture data.

Dataset	Adopted Technologies	# Subjects	# Activities	Activity	Monitored Body Part	# Seq
HMDB51 [6]	one monocular camera	-	51	General facial expressions, facial expression with object manipulation, general body movements, body movements with object interaction and body movements with human interaction	NA	6766
UCF101 [7]	one monocular camera	-	101	Human-object interaction, Body Motion Only, Human-human interaction, playing musical instrument and sports	NA	13,320
CMU MoBo [8]	six monocular cameras	25	1	Walking on a treadmill	NA	100
HumanEva I [9]	three monocular cameras, MoCap (six Vicon cameras)	4	6	Walking, Jogging, Gesturing, Throwing and Catching a ball, Boxing and Combo	Full Body	56
HumanEva II [9]	four monocular cameras, MoCap (twelve Vicon cameras)	2	1	Combo	Full Body	2
CMU Graphics Lab [10]	one monocular cameras, MoCap (twelve Vicon cameras)	>100	109	Human Interaction, Interaction with environment, Walking, physical activities and sports, situations and scenarios	Full Body	2605
HDM05 [11]	MoCap (twelve Vicon cameras)	5	>70	Walking, Object manipulation, Sports, Sitting and Lying and Other motions	Full Body	∼1500
KIT [12]	one monocular camera, MoCap (twelve Vicon cameras)	43	-	Walking, object manipulation, human-human interaction and other motions	Full Body	9727
CMU kitchen [13]	three monocular cameras, MoCap (twelve Vicon cameras), Audio (five microphones), five three-axis accelerometers and gyroscopes	31	5	Food preparation	Full Body	155
TUM Kitchen [14]	four monocular cameras, markerless MoCap, environmental RFID tags, magnetic sensors (detecting door/drawer opening)	4	4	Food preparation	Full Body	17
MHAD [15]	twelve monocular cameras, MoCap (eight Impulse cameras), two Microsoft Kinect (depth stream acquisition), Audio (four microphones), six three-axis accelerometers	12	11	Upper Limb actions, Lower Limb actions and Combined	Full Body	>647

**Table 2:** Principal ergonomic indices used to assess MSDs risk.

RULA	The Rapid Upper Limb Assessment method provides an overall score that takes into account postural loading on the whole body with particular attention to the neck, trunk, shoulders, arms and wrists. The overall score also takes into account the time the posture is held, the force used and the repetitiveness of the movement.
OCRA	The OCRA index is based on the ratio between Actual Technical Actions (ATA), obtained by analyzing the task, and Reference Technical Actions (RTA). The RTA value is obtained by taking into account the frequency and repetitiveness of movements, use of force, type of posture, recovery period distribution and additional factors such as vibration and localized tissue compression.
NIOSH	The NIOSH Lifting Equation is a tool used by occupational health and safety professionals to assess the manual material handling risks associated with lifting and lowering tasks in the workplace.

**Table 3:** Mean time, standard deviation and interquartile range of the execution time spent performing the working activities computed for the eight enrolled participants.

Execution Time [s]	Time [Mean ± std]	Time IQR
Handling goods	3.17 ± 0.98	3.33 (2.50, 5.26)
Hammering	3.62 ± 0.83	3.33 (3.03, 4.25)
Screwing	3.68 ± 0.82	3.58 (3.16, 4.25)

**Table 4:** Interquartile ranges of the maximum trunk rotations and mean and standard deviation of the trunk compensatory movements computed for all the trials recorded from the eight enrolled participants.

Trunk Angles [rad]	Handling Goods	Hammering	Screwing
max|tFE|	0.22 (0.19, 0.34)	0.34 (0.26, 0.53)	0.39 (0.28, 0.63)
max|tLB|	0.32 (0.25, 0.42)	0.21 (0.17, 0.25)	0.25 (0.21, 0.33)
max|tT|	0.17 (0.14, 0.29)	0.25 (0.15, 0.30)	0.25 (0.10, 0.28)
tFE	−0.06±0.12	0.13±0.19	0.15±0.25
tLB	0.17±0.10	−0.04±0.11	0.02±0.19
tT	−0.09±0.09	−0.08±0.07	−0.06±0.09

**Table 5:** Intra-subject variability.

max(V) [m]	Handling Goods	Hammering	Screwing
target 1	0.069314	0.12022	0.09883
target 2	0.049553	0.21264	0.066718
target 3	0.072194	0.24665	0.13023
target 4	0.055371	0.1774	0.070542
target 5	0.08587	0.072051	0.074852
target 6	0.07337	0.075214	0.063459
target 7	0.10273	0.048479	0.053727
target 8	0.12639	0.064105	0.065559
target 9	0.11809	0.10182	0.048844

## Data Availability

The WGD—Working Gesture Dataset built up during the current study will be publicly available after paper publication.

## Abbreviations

WGD:Working Gesture Dataset; MSDs: musculoskeletal disorders; Xip: xiphoid process; Jug: jugular; R_ac and L_ac: right and left acromion; R_e and L_e: right and left elbow; R_ul and L_ul: right and left ulna; R_rad and L_rad: right and left radius; R_h and L_h: right and left hand; R_f and L_f: right and left middle finger; C7: cervical vertebra C7; T10: thoracic vertebra T10; Sac: sacrum; R_asis and L_asis: right and left anterior superior iliac spine; sAA: sholder abduction/adduction; sFE: shoulder flexion/extension; sIE: shoulder internal/external rotation; eFE: elbow flexion/extension; wPS: wrist pronation/supination; wAA: wrist abduction/adduction; wFE: wrist flexion/extension; tFE: trunk flexion/extension; tLB: trunk lateral bending; tT: trunk torsion; V: variability; DoFs: degrees of freedom; IQR: interquartile range.
